# Editorial: Molecular Mechanisms of Thiol-Based Redox Homeostasis and Signaling in the Brain

**DOI:** 10.3389/fnagi.2021.771877

**Published:** 2021-10-13

**Authors:** Bindu Diana Paul, Milos R. Filipovic

**Affiliations:** ^1^Department of Pharmacology and Molecular Sciences, Johns Hopkins University School of Medicine, Baltimore, MD, United States; ^2^Leibniz-Institut für Analytische Wissenschaften – ISAS – e.V., Dortmund, Germany

**Keywords:** thiols, redox, neurodegeneration, signaling, aging, brain

Due to sulfur's ability to adapt to a wide range of oxidation states (−2 to +6), sulfur-centered chemistry played important role in the evolution of life on earth and remained conserved throughout all life forms (Paulsen and Carroll, 2013; Patel et al., 2015). Therefore, it is not surprising that thiols play a central role in maintenance of redox homeostasis in living systems. Thiols undergo myriad redox reactions which contribute to a rich array of signaling molecules. Cells are endowed with a multitude of sulfur containing molecules such as cysteine, homocysteine, lanthionine and taurine, peptides such as glutathione and gaseous signaling molecules such as hydrogen sulfide. Cysteine residues on proteins undergo the maximum number of posttranslational modifications, which include, but are not limited to nitrosylation, oxidation (from sulfenic and sulfinic to sulfonic acid), persulfidation, glutathionylation (Figure 1). Due to their nucleophilic nature, thiols also undergo electrophilic attack by naturally occurring electrophiles. These modifications play vital roles in cellular physiology and control responses to stress stimuli as well as normal cellular processes. As different modifications have different effects on protein structure and function, nature uses this redox-switching as an important mode of fine tuning of cellular signaling (Paulsen and Carroll, 2013).

**Figure 1:**
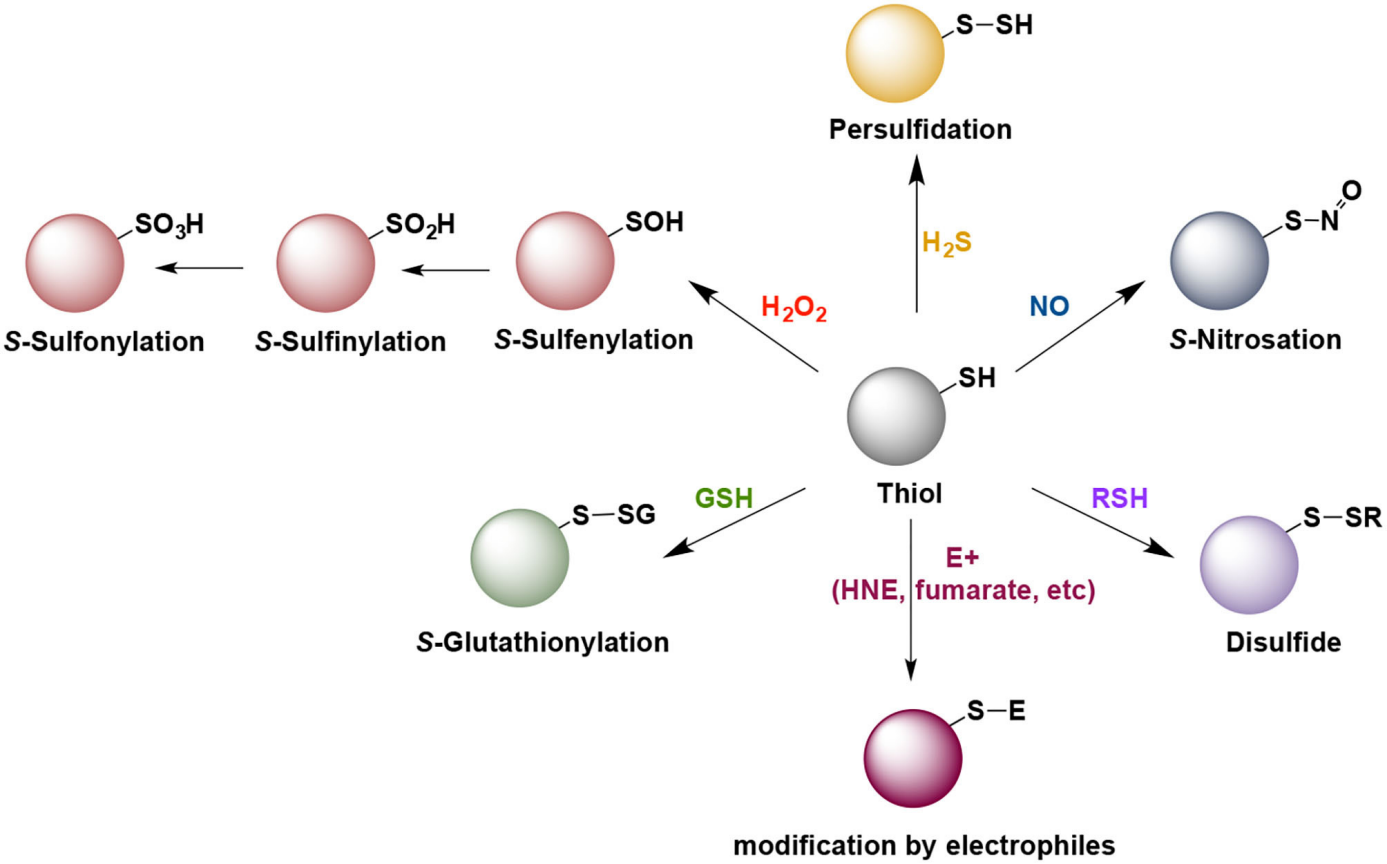
Some of the naturally occurring posttranslational modification of cysteine residues that play role in cell signaling and pathogenesis of neurodegenerative diseases. Note that with the exception of the H_2_O_2_ and intracellularly generated electrophiles (E^+^), all other reactions do not proceed directly and require an intermediate oxidation step.

Redox signaling is especially important in the brain, which is metabolically highly active. Disruption of redox signaling is a hallmark of several neurodegenerative diseases such as Alzheimer's disease, Huntington's disease, Parkinson's disease, Amyotrophic Lateral Sclerosis and Ataxias. Emerging evidence suggests that redox imbalance contributes to disease progression and pathophysiology of these diseases. Redox signaling in the brain has been relatively less studies as compared to that in peripheral tissues.

In this Research Topic, we bring together, a collection of articles that highlight the signal transduction cascades operating in the brain, their derangement in neurodegeneration and methods to quantify and detect these processes as well as points of therapeutic intervention.

Caused by the gasotransmitter nitric oxide, S-nitrosation of proteins is considered as an important signaling event used by the cells (Foster et al., 2009) but also as a cause and hallmark of neurodegenerative diseases (Nakamura et al., 2013). In her paper, Finelli provides an overview of the role of protein S-nitrosylation in brain aging and neurodegeration and discusses the potential of using redox-based therapeutic approaches for neurodegenerative conditions.

Similarly, to nitric oxide, another gasotransmitter, hydrogen sulfide, is implicated in regulating cellular function as well (Paul and Snyder, 2015; Filipovic et al., 2018). Produced through transsulfuration pathway from cysteine, this gaseous molecule also modifies cysteines, causing protein persulfidation. Paul discusses the role of dysregulated transsulfuration pathway in the pathogenesis of neurodegenerative diseases, focusing particularly on the recent discoveries about the role that disrupted H_2_S plays in Alzheimer's disease and the therapeutic benefits of H_2_S donors in a mouse model of Alzheimer's disease. On the other hand, Petrovic et al., summarize the current knowledge about protein persulfidation and its role in aging and aging-related neurodegenerative diseases, while particularly focusing on future direction that this field could take on.

Thiol modifications caused by intracellularly generated electrophiles is increasingly recognized as a mechanism utilized by cells to convey a stress stimulus (Parvez et al., 2018). One such molecule, fumarate, shows promising results in treatment of multiple sclerosis. In their article, Poganik and Aye provide an overview of lipid-derived electrophiles chemistry and of recent methodological advances to dissect these electrophile-signaling events in a protein/context-specific manner. Jové et al., on the other hand, focus on one specific electrophile-induced thiol modification, protein succination, caused by fumarate and discuss its role in brain signaling.

The issue also features an article, focusing on a well-established redox switch, Kelch-like ECH-associated protein 1 (Keap1). Keap1- Nuclear factor erythroid 2-related factor 2 (Nrf2) signaling axis is a validated and promising target for bolstering cellular defense and survival pathways. Hushpulian et al., discuss the potential off-target effects and their impact on future drug development originating from Keap1-targeting small molecules that function as displacement activators of the redox-sensitive transcription factor Nrf2.

## Author Contributions

Both authors wrote the manuscript.

## Conflict of Interest

The authors declare that the research was conducted in the absence of any commercial or financial relationships that could be construed as a potential conflict of interest.

## Publisher's Note

All claims expressed in this article are solely those of the authors and do not necessarily represent those of their affiliated organizations, or those of the publisher, the editors and the reviewers. Any product that may be evaluated in this article, or claim that may be made by its manufacturer, is not guaranteed or endorsed by the publisher.
